# Environmental tobacco smoke and children’s health: a bibliometric and altmetric analysis of 100 most cited articles

**DOI:** 10.1186/s12889-023-16242-1

**Published:** 2023-11-09

**Authors:** Sneha S. Patil, Naveen Puttaswamy, Sachin C. Sarode, Gargi S. Sarode, Smita S. Patil, Andres Cardenas, Rajesh Kumar Gandhirajan, Kalpana Balakrishnan

**Affiliations:** 1 Department of Environmental Health Engineering, Faculty of Public Health, Sri Ramachandra Institute of Higher Education and Research, Chennai, Tamil Nadu India; 2 Department of Pediatric and Preventive Dentistry, Dr. D.Y. Patil Dental College and Hospital, Dr. D.Y. Patil Vidyapeeth, Sant-Tukaram Nagar, Pimpri, Pune India; 3 Department of Oral Pathology and Microbiology, Dr. D.Y. Patil Vidyapeeth, Sant-Tukaram Nagar, Pimpri, Pune India; 4 Private Practitioner, Pune, India; 5 Department of Epidemiology and Population Health, Stanford School of Medicine, Stanford, CA USA; 6 Department of Human Genetics, Faculty of Biomedical Sciences, Sri Ramachandra Institute of Higher Education and Research, Chennai, Tamil Nadu India

**Keywords:** Altmetric, Bibliometric analysis, Dimensions, Environmental tobacco smoke, Child health, Most cited

## Abstract

**Background:**

Exposure to environmental tobacco smoke (ETS) is arguably the most ubiquitous and hazardous, even at very low levels, starting in early life. The objective of this study was to describe the state of research and future trends on ETS exposure and Children’s Health (CH) topics with bibliometrics and altmetrics.

**Methods:**

An electronic search was performed in Scopus database on January 31, 2023. Consensus was arrived on 100 most-cited articles by two reviewers. These papers were then cross matched with citations harvested from Web of Science (WoS) and Google Scholar. Altmetric Attention Score (AAS) and Dimension counts were also collected. Analysis and network visualization of authors, countries, and keywords were generated using VOSviewer software.

**Results:**

Among a total of 1107 articles published on ETS and CH, the 100 top-cited articles appeared in 54 journals, with Pediatrics (n = 12) contributing a maximum number of articles. The time period between 2000 and 2009 accounted for 44% of all publications. With respect to the research design employed across these studies, cross-sectional design took precedence over others accounting for approximately 40%. Predominantly, articles focused on childhood asthma; however, current research trends have shifted towards emerging fields such as children’s oral health and DNA methylation. Twitter, policy documents, and news outlets were the main platforms where outputs were discussed. The AAS was not associated with journal impact factor or access type. Weak correlations were observed between AAS and citation count in Scopus, WoS, and Google Scholar (r = 0.17 to 0.27) while a positive association existed between dimension count and the number of citations across all three databases (r = 0.84 to 0.98).

**Conclusion:**

This study demonstrates the evolution, digital dissemination and research hotspots in the field of ETS and CH, predicting the possible future research directions. High-quality studies with more specific exposure classification are warranted to better understand the relationship between ETS and CH.

**Supplementary Information:**

The online version contains supplementary material available at 10.1186/s12889-023-16242-1.

## Background

Exposure to environmental tobacco smoke (ETS) or second-hand smoke (SHS) significantly contributes to children’s morbidity and mortality. In 2004, it is estimated that ETS exposure led to approximately 603,000 premature deaths globally, with children accounting for 28% of these fatalities [1]. The literature is replete with compelling causality evidence between early life exposure (i.e., pregnancy to eight years) to ETS and numerous health outcomes in children. Parental smoking during pregnancy and exposure to ETS has been linked to impaired fetal growth, sudden infant death syndrome, preterm birth, low-birth weight, otitis media, respiratory illness, cardiovascular problems, neurodevelopmental effects, cancer and socio-behavioral inequities in adolescence and adult life [2–12].

Due to the growing burden of this condition, a formidable number of articles have been published. Subsequently, with massive literature it is an arduous task for researchers to narrow their search for a feasible number of high-quality papers. Citation-based indicators have traditionally been employed to assess this impact. Bibliometrics have been carried out on key scientific topics in various fields since 1987. Despite its widespread use, in recent years certain impetus in the foundation has emerged, challenging its position as the leading indicator of research impact. Use of alternative metrics has invoked several studies to address the realm of other indicators [13]. Altmetrics has recently emanated as a web-based screening tool that evaluates the individual influence of an article through online attention. They can include (but are not limited to) citations on Wikipedia and in public policy documents, patents, discussions on research blog, multi-media sites (YouTube), online reference managers like Mendeley, CiteULike, and social networks (Facebook, Twitter) [14]. Altmetric computes an Altmetric Attention Score (AAS) formulated on its mention in these platforms, which are ascribed specific weights and amalgamated into a single index [15].

To date, there has been no bibliometric analysis on ETS and children’s health (CH) despite its growing public health concern, exploding publication records, and mounting scientific evidence. In addition, no comprehensive study has assessed the relationship between traditional and alternative metrics on this topic. Thus, this study aimed to analyze the 100 most cited articles using bibliometric and altmetric methods to provide an overview of the current research on ETS exposure and its impact on children’s health.

## Methods

A comprehensive search of the Scopus database was performed on January 31, 2023. The search terms used were “environmental tobacco smoke,” “secondhand tobacco smoke,” “passive smoking,” “involuntary smoking” and “child health”. A total of 1,107 articles were retrieved, without restrictions on publication date or language. The first 300 items were exported in a CSV (comma-separated values) file format. The titles and abstracts of studies identified from the search were scanned independently by two authors and if necessary, the full-text articles were analyzed. Every paper was screened in consonance with the inclusion criteria: (a) articles focused on any aspect of ETS and CH (b) original research, case series/reports, and reviews. Papers not related to ETS and CH were excluded. A total of 189 articles were scrutinized and 89 were discarded in accordance with the selection criteria. The 100 included articles were then ranked according to the decreasing number of their citations. When articles had equal citation counts, the paper published recently was graded higher.

### Evaluation with other data sources

The selected articles were cross-examined with the citation data from Web of Science (WoS) Core Collection and Google Scholar to compare the number of citations. The Altmetric bookmarklet was added to the Google Chrome browser toolbar. The article under consideration was accessed on PubMed and evaluated using the ‘Altmetric It’ tool, with scores retrieved from the resulting doughnut popup. Further details regarding Altmetric scores were obtained by clicking on a ‘click for more details’ button. Dimensions citation count was also captured through the hyperlink.

### Data extraction

Two review authors assessed the selected articles and extracted the citation attributes (title, authors, country, authors affiliations, funding sources, year of publication, citations, the title of the scientific journal, impact factor, quartile scores). Furthermore, the study design, the topic addressed, and article access (i.e., subscription for access vs. free access) was discerned.

### Bibliometric network

VOSviewer (version 1.6.19, Leiden University, Netherlands) was used to create co-authorship, countries, and keyword co-occurrence networks. For the co-occurrence analysis of ‘all keywords,’ items that appeared in singular and plural form, like ‘risk factor’ and ‘risk factors’ and differed by a hyphen, such as ‘preschool’ and ‘pre-school’ were selected from the CSV file and combined. Irrelevant keywords were excluded from the analysis.

### Data analysis

The Journal Impact Factor (JIF) in 2022 was accessed in the WoS’s Incites Journal Citation Reports. Articles were also tabulated using the recent edition of the International Classification of Diseases (ICD-11). Descriptive statistics were used to describe the primary data set. Pearson correlation coefficient (r) was used to evaluate the relationship between citation counts for individual papers, AAS, and Dimensions count. A p-value </=0.05 was considered statistically significant. Statistical analysis was performed using the Statistical Package for Social Sciences version 29.0.0 (IBM Corporation, Armonk, NY, USA).

## Results

### Citation counts

Additional file 1 shows the ranking of the 100 most-cited publications. The top-cited articles accrued a total of 10,463 (Scopus), 6305 (WoS), and 17,149 (Google Scholar) citations. DiFranza et al.’s article “Prenatal and Postnatal Environmental Tobacco Smoke Exposure and Children’s Health” published in the Pediatrics journal (2004) was the most cited paper, with 641 (Scopus), 549 (Web of Science), and 984 (Google Scholar) citations and a mean citation density of 90.4. The article “Prenatal Tobacco Smoke Exposure affects global and Gene-specific DNA methylation” ranked second, with 484 (Scopus), 435 (Web of Science), and 675 (Google Scholar) citations, and a mean citation density of 86.93. Thirty-seven articles were not detected by WoS.

### Publication characteristics

#### Journal characteristics and year of publication

**Fig. 1:**
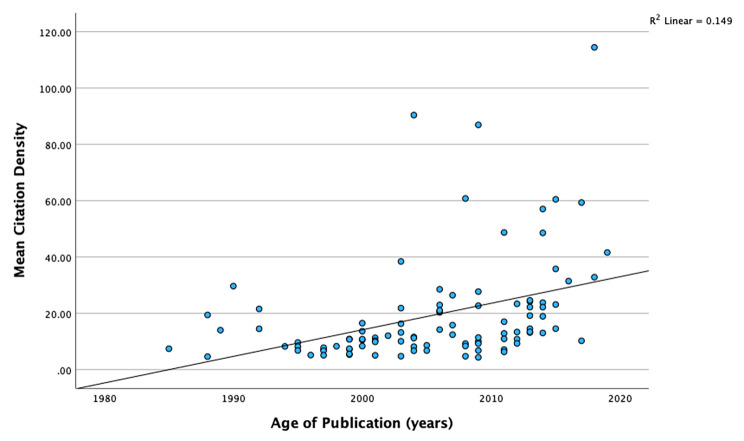
Association between age of publication and mean citation density

The articles recognized in the search came from 54 journals, amid them, 42 were positioned in the first quartile, nine and three in the second and third quartile respectively (Additional file 2). Twenty journals were edited in the United Kingdom and 15 in the United States. A maximum number of publications were contributed by the Pediatrics journal (n = 12), followed by the American Journal of Epidemiology and the International Journal of Epidemiology (n = seven each). Thirty-nine journals provided only a single paper. The JIF ranged from 0 to 93.333 (mean 8.48 ± 13.18). Free full-text was accessible (Open Access) for 22 articles, while 78 papers required a subscription. The distribution of citation counts significantly differed between freely available studies (median − 99.5, SD 50.38, total number of citations − 2350) and restricted access papers (median − 71.5, SD 97.64, total number of citations − 8113). The years 2009 and 2013 had the highest number of articles, eight and seven respectively. Our findings revealed that the number of papers published on ETS and CH reached its peak between 2000 and 2009 (44%). Twenty-three articles were published between 1985 and 1999, while 33 papers between 2011 and 2019. There was a positive association between the mean citation density and age of publication, however, it was weak (r = 0.08; p = 0.04) as shown in Fig. 1.

#### Authors, country, institution, and funding source distribution

**Fig. 2:**
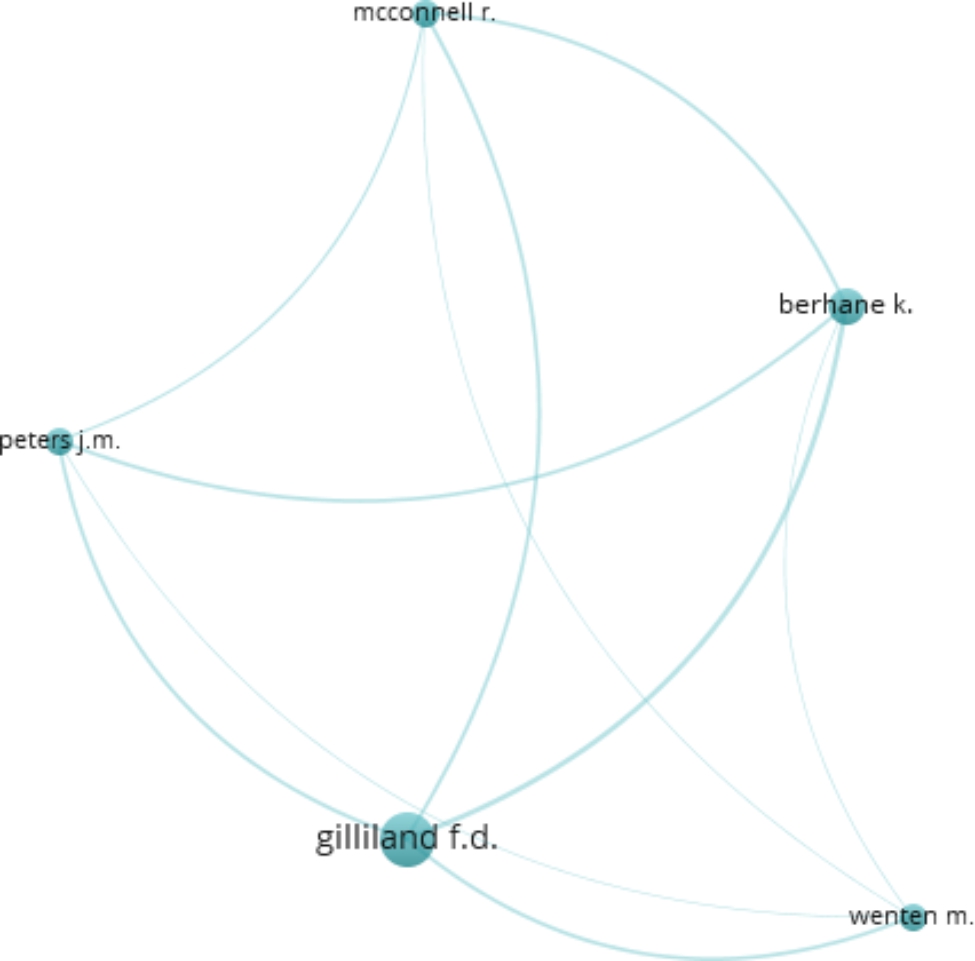
Coauthor contribution in the top-cited papers

**Fig. 3:**
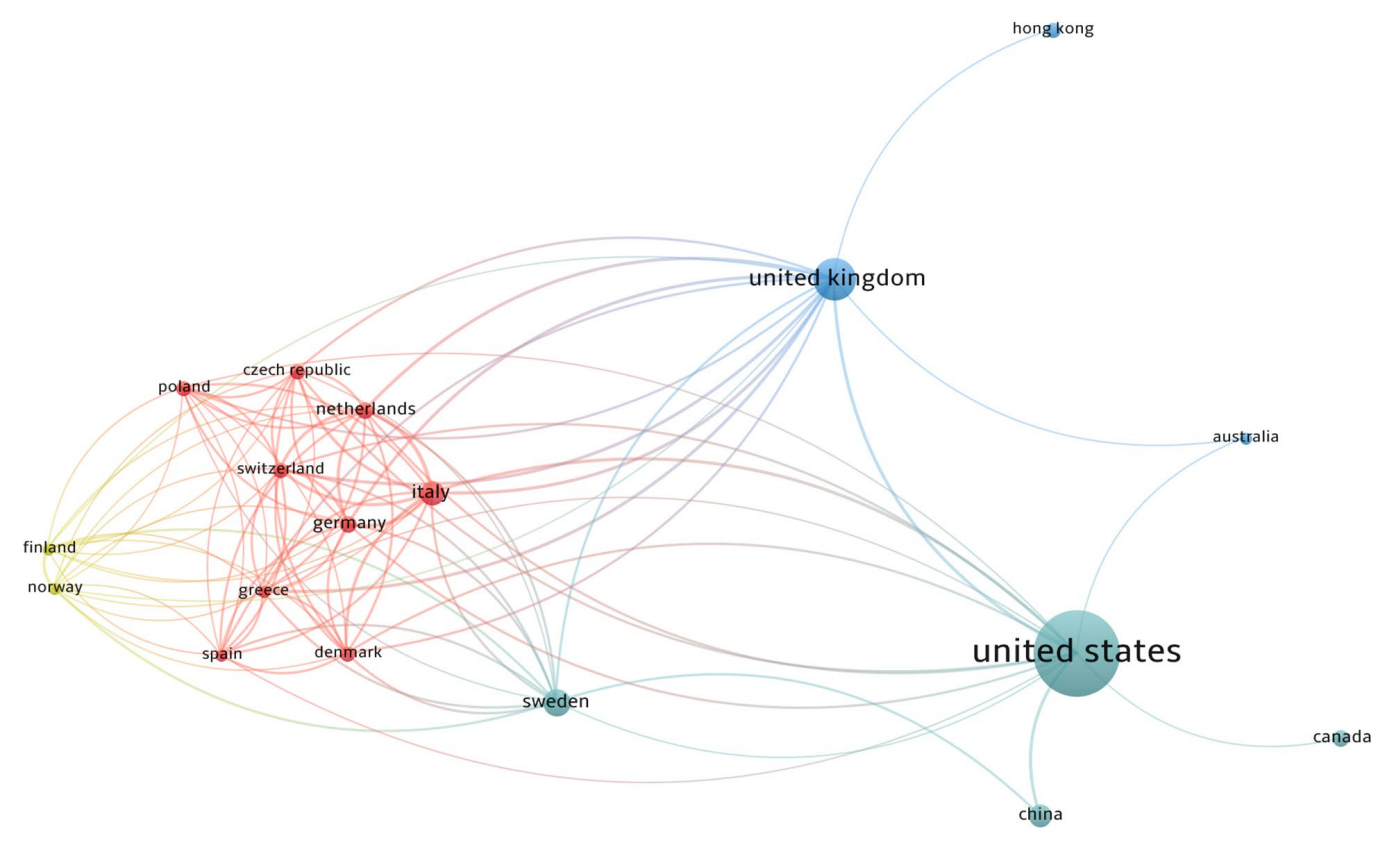
Visualisation network of international collaboration for ETS and CH publications

One hundred and sixty researchers contributed to the top-cited articles. The articles were published by Frank D. Gilliland (six articles; 1029 citations), Martin Weitzman (five articles; 1413 citations), Kiros Berhane, Jouni J.K. Jaakkola, and Lam Tai Hing (four articles each; 497, 322, 272 citations respectively). Sixty-six publications were authored by one to six researchers, whereas 34 studies had seven to forty authors. A co-authorship relation was also developed (Fig. 2). Fifteen of the 51 most productive authors were integrated into the confederation network. This was led by Frank D. Gilliland and Kiros Berhane involving five authors each. The influential articles emerged from 38 diverse nations. The United States contributed to majority of the publications (46 articles; 5420 citations), followed by the United Kingdom (17 articles; 1537 citations), Sweden (9 articles; 682 citations), Italy (8 articles; 1064 citations), and China (7 articles; 493 citations). Figure 3 displays the collaboration network of countries that were drawn to meet the threshold of a minimum of three publications. The United States, the United Kingdom, and Sweden had a substantial number of international collaborations. The countries were categorized into clusters, with each cluster depicted by color. The node size is an indicator of the number of papers published by each nation. The publications co-authored are represented by the joining lines, with thicker lines signifying a stronger link between the two countries. The University of California, Berkeley allied the most papers (n = 8), followed by seven articles from the Harvard TH Chan School of Public Health, and six articles from Keck School of Medicine, University of Southern California. The National Institute of Environmental Health Sciences was the topmost organization to fund 15 studies, whereas the National Heart, Lung, and Blood Institute and the National Cancer Institute sponsored six and five studies respectively.

#### Research design, health outcome addressed and keyword co-occurrence network analysis

**Fig. 4:**
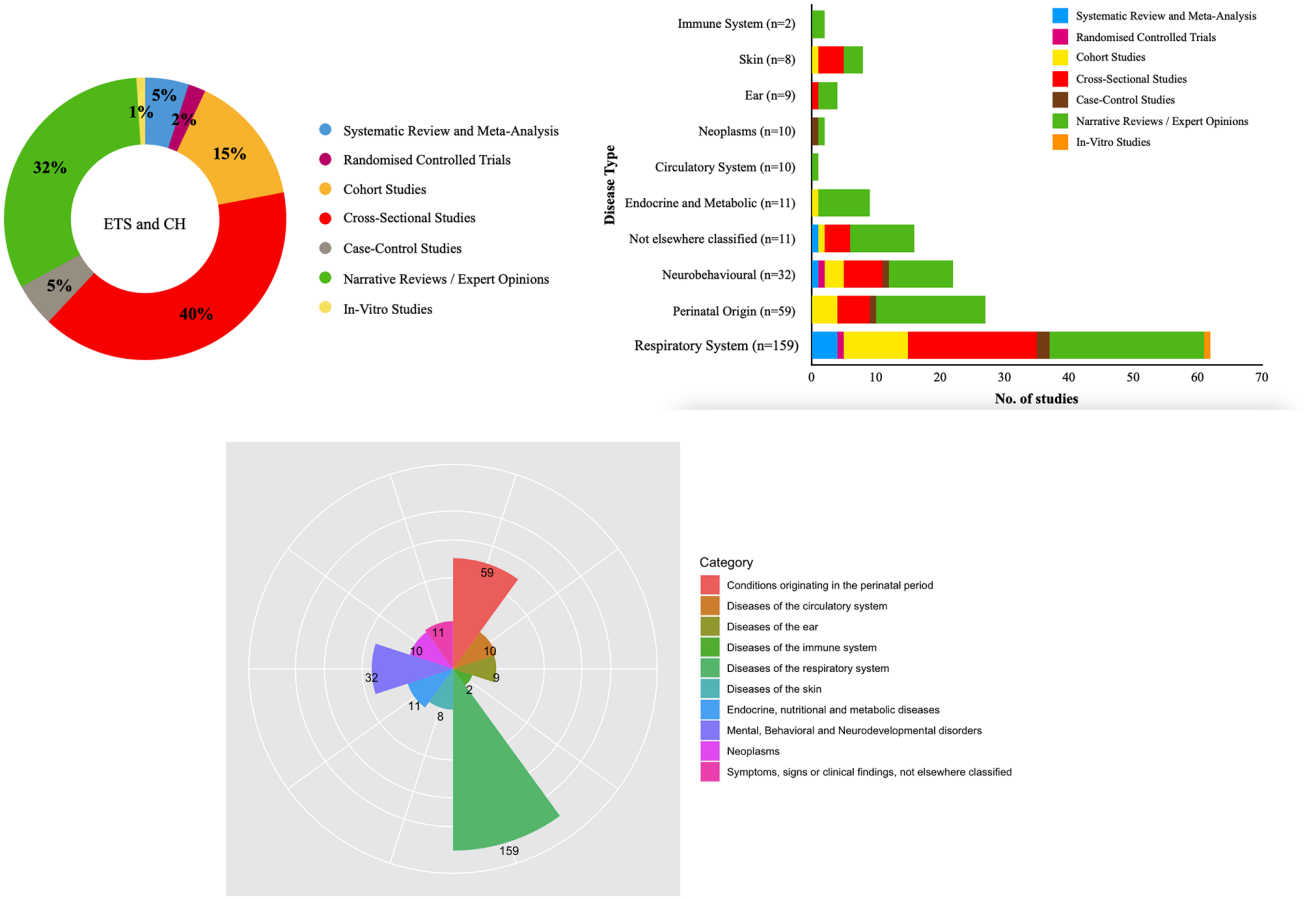
Distribution of (**a**) articles by study design (**b**) disease type by study design (**c**) disease type based on ICD-11

**Fig. 5:**
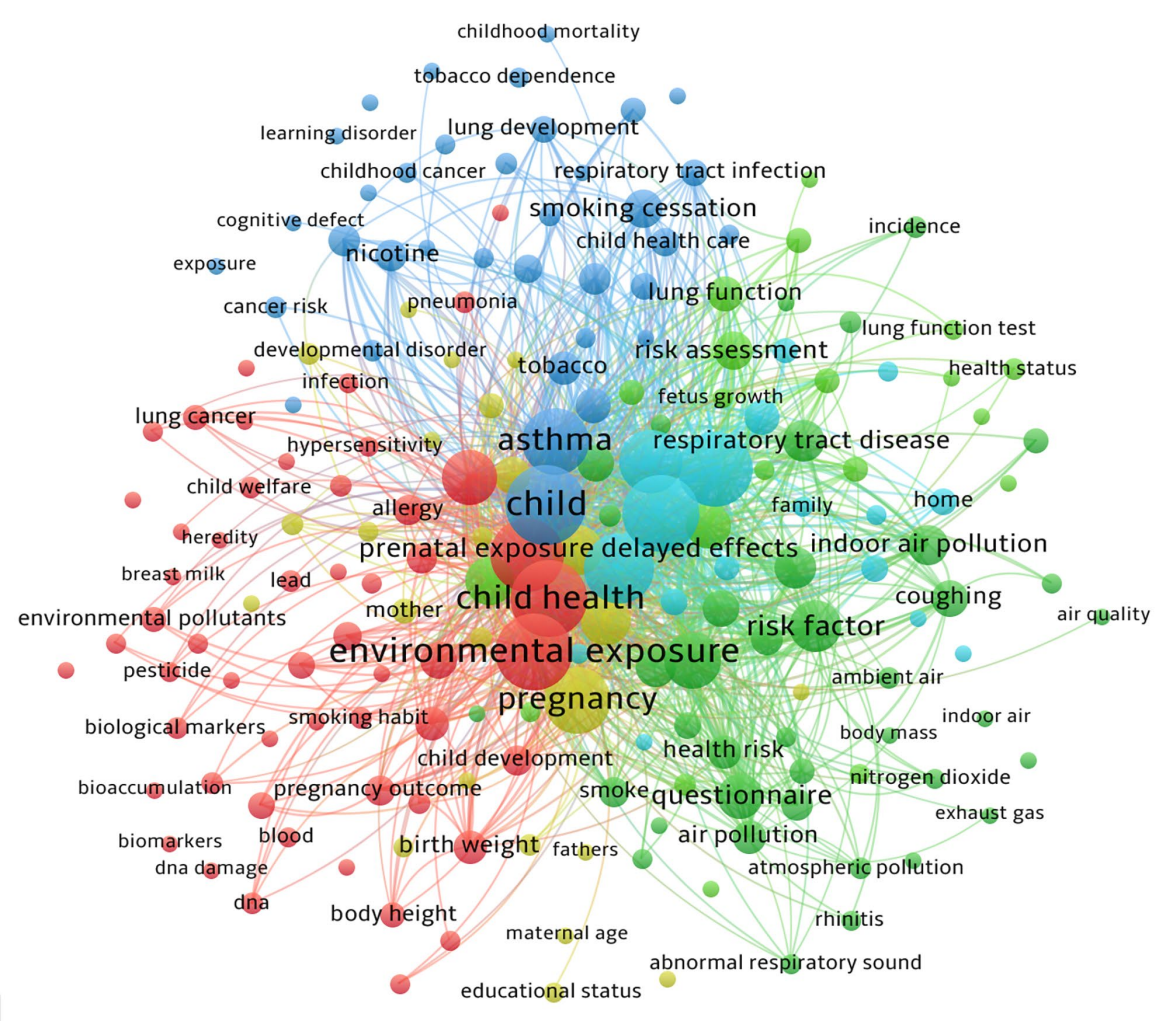
Keyword co-occurrence map of the most-cited articles

Cross-sectional studies were observed to be the most prevalent study design accounting for 40% (3623 citations). Narrative reviews, cohort studies, systematic reviews, and case-control studies constituted about 32% (3945 citations),15% (1333 citations), 5% (602 citations), and 5% (806 citations) respectively (Fig. 4a). The assessment of health outcomes addressed based on the International Classification of Diseases (ICD-11) disclosed that respiratory diseases (n = 159) were the most frequently cited. This was followed by conditions originating in the perinatal period (n = 59), neurobehavioural disorders (n = 32), endocrine and metabolic diseases (n = 11), childhood cancer, and cardiovascular effects (n = 10 each) (Fig. 4b). Figure 4c demonstrates the disease distribution by study design. A total of 1442 keywords that formed five clusters were detected in the present analysis. Figure 5 demonstrates the keyword co-occurrence relation. The most prominent node was “environmental exposure” which emerged 61 times. This was followed by “passive smoking” (60), “child health” (59), and “tobacco smoke pollution” (58). Surprisingly, the keyword “environmental tobacco smoke” and “secondhand smoke” appeared only a mere 13 and three times respectively. Cluster 1 in red mainly included “child health,” “environmental exposure,” “maternal exposure,” “biomarkers,” “birth weight,” “allergy,” “pneumonia,” and “DNA damage.” It reflected the researchers’ focus on how ETS exposure affects children’s health. Cluster 2 in green constituted “air pollution,” “atmospheric pollution,” “ambient air,” “smoke,” “home environment,” and “questionnaire.” It investigated the components and discussed the conditions in which ETS may be harmful to public health. Cluster 3 in blue primarily covered “adverse outcome,” “low birth weight,” “cognitive defect,” “childhood obesity,” “childhood cancer,” “respiratory tract infections,” and “middle ear disease.” Cluster 3 indicated they were interested in a range of child health problems, a sub-theme of Custer 1. Cluster 4 in yellow contained “maternal smoking,” “maternal age,” “educational status,” “pregnancy,” “hypertension,” and “father.” This emphasized the role of parental influence on ETS. While cluster 5 which discussed the potential mechanism of ETS comprised “pathophysiology,” “risk assessment,” and “respiratory function test.” From 2010 to 2019, “nicotiana tabacum,” “rhinitis,” “lower respiratory tract infections,” “birth weight,” “DNA,” “child behavior disorders,” and “fetal development” have started to draw attention.

### Altmetric indicators

The total AAS for the papers was 937 (median = 3) with individual values ranging from 0 to 149. Thirty-two percent of the top-cited articles had no AAS. The outputs were mostly discussed on Twitter (median = 2; range from 0 to 25), policy documents (median = 1; range from 0 to 6), and news outlets (median = 2; range from 0 to 19). Uploaded videos and patents were much less significant. Strzelak’s ‘Tobacco smoke induces and alters immune responses in the lung triggering inflammation, allergy, asthma, and other lung diseases: A mechanistic review’ was the most popular online article (AAS = 149). The breakdown of the AAS revealed this research was cited in 19 new outlets, seven tweets, one blog post, one Facebook page, one Wikipedia page, one video uploader and referenced 431 times in Mendeley (Additional file 3). The AAS was not significantly associated with the JIF (r = -0.01, p > 0.05). The AAS was also not significantly correlated between articles published in Q1 journals compared to those published in Q2 and Q3 journals. Similarly, no significant difference was noted in AAS between articles with unrestricted access and those that require a subscription (p > 0.05). There was a weak correlation between the AAS and citation counts in Scopus (r = 0.17, p = 0.16), WoS (r = 0.27, p = 0.02), and Google Scholar (r = 0.17, p = 0.16). Conversely, a positive association was found between the dimensions citation count and the number of citations in Scopus (r = 0.98, p < 0.000), WoS (r = 0.84, p < 0.000), Google Scholar (r = 0.94, p < 0.000). It should be emphasized that a Pearson’s correlation analysis was carried out on only those papers with a greater than one Altmetric score and Dimension count (Additional file 4).

## Discussion

Analysis of the top 100 cited articles on exposure to ETS and its impact on children’s health provides a varied yet persuasive read. This study links conventional indicators of bibliometrics with the modern digital dissemination measures for studies relating to ETS and CH. Currently, they appear to have discrete but reciprocal parts in assessing the broadcasting and influence of these publications.

One of the most striking features of the list is papers that appeared in journals with a low IF garnered substantial citations, whereas articles emerging in high-IF journals received limited references. The Pediatrics journal had the maximum number of articles (n = 12, JIF 9.703), whilst the British Medical Journal with maximal IF 93.333, presented only four studies. This suggests that citations are more dependent on the content and scientific ‘popularity’ of the research topic among researchers than the JIF. This study observed 33 articles with 100 or greater citation counts, thus making them citation classics [16]. They were cited between 100 and 641 times when the evaluation was employed with Scopus. A comparison across multiple data sources revealed variations in citation numbers; citations varied between ranges of 41–641 (Scopus), 35–549 (WoS), and 38–984 (Google Scholar). This difference underscores the purport of selecting a relevant scientometric database. Scopus provides a wide breadth of journals (n = 12,850) than WoS (n = 8,700) and quicker citation analysis. WoS and Google Scholar were not used as benchmark data sources for numerous grounds. In WoS, missing and incorrect references are major issues. Google Scholar includes citation data from books, preprints, theses, and dissertations which may influence the evaluation of the top publications [17]. Interestingly, two highly cited papers by Weitzman M on “Maternal Smoking and Childhood Asthma” and “Maternal Smoking and Behavior Problems of Children” were only found in Scopus and Google Scholar but not in WoS. It is worth noting that while citation counts do not delineate the study quality, it imitates its acclaim within the research community and impact on shaping discussions, controversies, practice guidelines or further investigations [18].

Although older literature is likely to be more frequently referenced, we observed a significant inclination towards recently published articles, with 33 papers that were released within the last decade. This can be attributed in part to the increasingly prominent role of digital platforms in evidence-based medicine, enabling manuscripts to explore novel concepts and guide future research trajectories. Interestingly, over the years the number of co-authors has risen substantially, with a preponderance of publications having more than three authors. A possible explanation could be increased awareness and interest among researchers of numerous institutes and countries about the potential benefits that studies in the purview of ETS could provide in children’s health. The average number of researchers per publication was 6.19. Frank D. Gilliland, a leading investigator in air pollution research, respiratory health, and gene-environment interactions, was on top of the list with six articles and a mean citation density of 29.31. In this analysis, it was observed that authors tend to collaborate quite frequently with authors affiliated with the same university or country. Frank D. Gilliland and Kiros Berhane had maximum collaborations with researchers. More coalition amongst investigators can be expected in the future.

As evidenced by the present study and in concordance with other bibliometric studies in varied fields, the majority of studies stemmed from academic institutes in the United States. Countries with a stronger economic background are inclined towards biomedical research, perhaps due to better scientific resources and funding. Despite the high prevalence and fatalities associated with exposure to ETS among children in low- and middle-income nations, there were limited population-based investigations performed within these regions. This study recognized a trend towards collation between the United States and several other nations, including the United Kingdom, Sweden, Italy, Netherlands, Poland, Germany, Switzerland, Greece, Denmark, Spain, China, Canada, Australia. Notably, among the top 100 cited articles, there were only two randomized controlled trials and five systematic reviews, while narrative reviews dominated with a count of 32. It is important to acknowledge the challenges of conducting randomised controlled trials for hazardous exposures like ETS even when trying to implement beneficial interventions. Furthermore, with the colossal size of publications, researchers may incline to consolidate and synthesize the existing information on a topic in the form of a literature review. Though Cochrane reviews have been internationally acclaimed as the highest level of the evidence base, they could ensure only one position in this study. A plausible explication of the lower citation counts could be that they are yet to attain a substantial age of publication. Fifty-five percent of the research papers were observational (cohort or cross-sectional). This finding could be attributed to the relatively lower resource requirements and costs associated with these study designs. Fundamental explorations in the etiopathogenesis of ETS have emanated from this study design. As the evidence-based philosophy is being propagated globally, it is essential to prioritize meticulously planned high-quality clinical studies on ETS and CH. Urgent attention must be directed towards conducting large longitudinal studies that span from preconception until childhood to gain a better understanding of how exposure to ETS impacts subclinical childhood health outcomes, such as neuropsychologic impairments. Additionally, large-scale case-control studies are required to investigate gene-environment interactions for relatively uncommon diseases like childhood malignancies. However, there are two challenges present within this field: exposure misclassification and statistical methodologies required for dealing with intricate interactions comprising multiple dimensions. Future research efforts could immensely benefit from using archives of exposure biomarkers which hold crucial information on prenatal and childhood determinants of adult diseases. While the primary target organ for ETS exposure is the lungs it comes as no surprise that a considerable number of studies (n = 31) focussed on respiratory outcomes such as asthma, wheezing, pneumonia, acute respiratory infections, and lower respiratory infections. There exists a substantial amount of evidence to support the causal relationship between exposure to ETS and respiratory ailments as compared to other conditions. There was a scarcity of studies assessing the association between ETS and atopic eczema (n = 9) or otitis media (n = 8). Similarly, the number of articles about ETS and snoring, and obesity were also low. The relationship between ETS and childhood dental caries is an area of research that is expanding. Furthermore, the expeditious growth of DNA methylation has aided the ranking of epigenetic papers, a part of Precision Environmental Health, to gain notable traction in the past ten years. It is paramount to take cognizance of the detrimental effects of ETS on childhood illnesses that could potentially influence their health trajectory throughout adult life. A collaborative effort between communities, healthcare professionals and government bodies at all levels must be pursued to explore novel solutions within the realm of children’s environmental health. Thereby, successfully translating and communicating research findings into actionable interventions. Finally, the process of triangulation of evidence by means of reviews and pointing sources of bias in different study designs can help strengthen the degree of causality from multiple study designs [19].

The evaluation also focused on both the authors’ chosen keywords and those indexed in the papers. The commonly used term “human” was frequently observed, along with gender-specific words such as “male” and “female.“ Thus, when searching for papers related to ETS and CH, employing generic keywords may result in a more compendious search.

The conventional citation-based indicators do not assess the social media realm. As highlighted in additional file 3 the highest altmetirc score was displayed by a mechanistic review of tobacco smoke altering the immune responses in the lung triggering inflammation by Strzelak et al. (2018). This article was broadcasted through various news outlets and tweets; nineteen and seven times, respectively. On the contrary, the second article ‘Housing Characteristics and Children’s Respiratory Health in the Russian Federation’ published in 2004, was broadcasted by seven agencies but received low Twitter dissemination. From this study, we see the growth in Twitter and news outlets’ distribution of research cognates by a regress in blogs, CiteULike, and Facebook’s use to exchange scientific literature. Conjectures can be derived if these configurations demonstrate an alteration in the overall repute or if more distinct role changes amid social network types have led to this makeshift; however, further investigation is warranted. The percentage of papers with the maximal AAS suggests a huge diversity among the journals with 8% published in the Pediatrics journal followed by 5% each in the International Journal of Epidemiology and Environmental Research journal.

The relationship between the citations in WoS, Scopus, Google Scholar, and the observed AAS was poor. The lack of relation between the number of citations and AAS can be elucidated either by the varied nature of the items which have been taken for estimation or the distinct responses of a scholar/populace to a publication. A strong correlation was noted between Dimensions count and Scopus, WOS, and Google Scholar citation count. Dimensions count may be paramount since it can partially overcome the bias of Altmetric owing to the inconsistent features of social networks [20]. The AAS of environmental tobacco smoke and child health articles was not significantly correlated with the quartile of the journals. Similar results have been stated by other studies [21, 22]. Altmetric outcomes need to be conferred with prudence since the articles published before the burgeoning of the social media landscape may be under-represented [23]. Altmetrics evaluates the immediate influence of an article, in contrast to the traditional metrics where papers may take more than a decade to attain maximal citations [22]. Our findings displayed social media mentions reached a peak in the first five years after publication, this is in accordance with similar studies [21, 24].

Besides the aforementioned time delay in citations, the results of the study should be expounded with caution. Bibliometric and altmetric analyses have numerous inherent limitations. Firstly, landmark studies, over time, achieve fewer citations as their findings are absorbed into current knowledge without the necessity for referencing. This is referred to as “obliteration by incorporation” [16]. To mitigate this, we discerned articles by citation density. Second, self-citations can have an impact on citation counts. In this analysis, however, a major variance between the total number of citation counts and citations was not reported after excluding self-citations. Third, only articles published since July 2011 are picked up by Twitter. Also, the Bookmarklet works only on PubMed, arXiv, or Google Scholar pages containing a DOI [14]. Hence, the probability of influential articles not being cited by social media scientometrics cannot be ruled out. Fourth, altmetrics recognize the level of online activity of research without distinguishing between the publicity or the research output quality [15]. Fifth, altmetrics weight allocation in the generation of scores is related to the developer’s inference about their anticipated goal for every media platform [24]. Thus, there may be an imbalance in the contribution of diverse sources to AAS. Sixth, researchers can “game” the system by generating added mentions for their projects on a social forum [25]. This type of manipulation bias was improbable in the present study as Altmetric Explorer was used as a search engine.

Alternative metrics are in their early stages, and there is meager data about the elements of social platforms to certainly elucidate a definite association amidst novel metrics and bibliometrics. It is ambiguous if media presence leads to higher citations or if aspects that steer greater citation counts lead to increased social networking activity. Although the social web may have some cogency on the distribution of an article, alternative metrics ought to be employed alongside traditional bibliometric measures for assessing research impact comprehensively. Future investigations should explore methods to construct a comprehensive stratagem that integrates both citation-based and social media-based indicators for evaluating research outcomes.

## Conclusion

This article provides scientometric and digital dissemination of ETS and CH research between 1985 and 2019. Numerous publications providing strong evidence of causality linking ETS exposure to several pediatric illnesses were noted. However, additional long-term studies of ETS exposure and CH are needed particularly in low- and middle-income countries to provide more precise estimates of these effects. A poor association between the citations in Scopus, WoS, Google Scholar, and the AAS existed, whilst the Dimensions score had a strong relationship with the data sources. To enhance the social influence of research on ETS and CH, sharing research outputs through social media platforms should be encouraged by editors and publishers to reach wider audiences including researchers, academicians, and policy analysts.

### Electronic supplementary material

Below is the link to the electronic supplementary material.

Supplementary Material 1

Supplementary Material 2

Supplementary Material 3

Supplementary Material 4

## Data Availability

All data are contained within the manuscript and its additional files.

## Abbreviations

ETS: Environmental Tobacco Smoke
CH: Children’s Health
WoS: Web of Science
AAS: Altmetric Attention Score
JIF: Journal Impact Factor
