# Supplementary material for: Environmental tobacco smoke and children’s health: a bibliometric and altmetric analysis of 100 most cited articles
Source: BMC Public Health. 2023 Nov 9;23:2208. doi: 10.1186/s12889-023-16242-1 (PMC10634132; doi:10.1186/s12889-023-16242-1)
Supplement: Supplementary file 1 — Supplementary Material 1 [file 12889_2023_16242_MOESM1_ESM.docx]

S1 Table - Citation count of the 100 most‐cited publications

| **Authors** | **Title** | **Year** | **Citation count (Scopus)** | **Citation count (Web of Science)** | **Citation count (Google Scholar)** | **Mean citation density** |
| --- | --- | --- | --- | --- | --- | --- |
| DiFranza JR, Aligne CA, Weitzman M | Prenatal and Postnatal Environmental Tobacco Smoke Exposure and Children's Health | 2004 | 641 | 549 | 984 | **90.4** |
| Breton CV, Byun HM, Wenten M, Pan F, Yang A, Gilliland FD | Prenatal tobacco smoke exposure affects global and gene-specific DNA methylation | 2009 | 484 | 435 | 675 | **86.93** |
| Weitzman M, Gortmaker S, Klein Walker D, Sobol A | Maternal smoking and childhood asthma | 1990 | 360 | - | 649 | **29.67** |
| Wigle DT, Arbuckle TE, Turner MC, Bérubé A, Yang Q, Liu S, Krewski D | Epidemiologic evidence of relationships between reproductive and child health outcomes and environmental chemical contaminants | 2008 | 335 | 296 | 539 | **60.79** |
| Hofhuis W, de Jongste JC, Merkus PJFM | Adverse health effects of prenatal and postnatal tobacco smoke exposure on children | 2003 | 263 | 237 | 465 | **38.42** |
| Weitzman M, Gortmaker S, Sobol A | Maternal smoking and behavior problems of children | 1992 | 263 | - | 427 | **21.56** |
| Strzelak A, Ratajczak A, Adamiec A, Feleszko W | Tobacco smoke induces and alters immune responses in the lung triggering inflammation, allergy, asthma and other lung diseases: A mechanistic review | 2018 | 234 | 224 | 378 | **114.44** |
| Jones LL, Hashim A, McKeever T, Cook DG, Britton J, LeonardiBee J | Parental and household smoking and the increased risk of bronchitis, bronchiolitis and other lower respiratory infections in infancy: Systematic review and meta-analysis | 2011 | 196 | 193 | 373 | **48.71** |
| Corbo GM, Fuciarelli F, Foresi A, De Benedetto F | Snoring in children: Association with respiratory symptoms and passive smoking | 1989 | 186 | - | 305 | **14.02** |
| Pattenden S, Antova T, Neuberger M, Nikiforov B, De Sario M, Grize L, Heinrich J, Hruba F, Janssen N, LuttmannGibson H, Privalova L, Rudnai P, Splichalova A, Zlotkowska R, Fletcher T | Parental smoking and children's respiratory health: Independent effects of prenatal and postnatal exposure | 2006 | 177 | 149 | 287 | **28.53** |
| Banderali G, Martelli A, Landi M, Moretti F, Betti F, Radaelli G, Lassandro C, Verduci E | Short and long term health effects of parental tobacco smoking during pregnancy and lactation: A descriptive review | 2015 | 171 | 166 | 318 | **60.48** |
| Etzel RA, Pattishall EN, Haley NJ, Fletcher RH, Henderson FW | Passive smoking and middle ear effusion among children in day care | 1992 | 163 | - | 301 | **14.5** |
| Zar HJ, Ferkol TW | The global burden of respiratory disease - Impact on child health | 2014 | 159 | 152 | 276 | **48.56** |
| Li YF, Gilliland FD, Berhane K, McConnell R, Gauderman WJ, Rappaport EB, Peters JM | Effects of in utero and environmental tobacco smoke exposure on lung function in boys and girls with and without asthma | 2000 | 157 | - | 239 | **16.5** |
| Julvez J, RibasFitó N, Torrent M, Forns M, GarciaEsteban R, Sunyer J | Maternal smoking habits and cognitive development of children at age 4 years in a population-based birth cohort | 2007 | 156 | 145 | 245 | **26.42** |
| Gilliland FD, Berhane K, Li YF, Rappaport EB, Peters JM | Effects of early onset asthma and in utero exposure to maternal smoking on childhood lung function | 2003 | 151 | 135 | 263 | **21.85** |
| Cheraghi M, Salvi S | Environmental tobacco smoke (ETS) and respiratory health in children | 2009 | 144 | 125 | 230 | **27.71** |
| Wigle DT, Arbuckle TE, Walker M, Wade MG, Liu S, Krewski D | Environmental hazards: Evidence for effects on child health | 2006 | 140 | 116 | 235 | **22.98** |
| McEvoy CT, Spindel ER | Pulmonary Effects of Maternal Smoking on the Fetus and Child: Effects on Lung Development, Respiratory Morbidities, and Life Long Lung Health | 2017 | 138 | 124 | 236 | **59.33** |
| Neri M, Ugolini D, Bonassi S, Fucic A, Holland N, Knudsen LE, Šrám RJ, Ceppi M, Bocchini V, Merlo DF | Children's exposure to environmental pollutants and biomarkers of genetic damage: II Results of a comprehensive literature search and meta-analysis | 2006 | 133 | 108 | 197 | **20.33** |
| Schoeters G, Den Hond E, Zuurbier M, Naginiene R, Van Den Hazel P, Stilianakis N, Ronchetti R, Koppe JG | Cadmium and children: Exposure and health effects | 2006 | 128 | 112 | 208 | **20.74** |
| Chang JS, Selvin S, Metayer C, Crouse V, Golembesky A, Buffler PA | Parental smoking and the risk of childhood leukemia | 2006 | 128 | 120 | 211 | **21.05** |
| Pinkerton KE, Joad JP | The mammalian respiratory system and critical windows of exposure for children's health | 2000 | 124 | - | 204 | **13.66** |
| McConnell R, Shen E, Gilliland FD, Jerrett M, Wolch J, Chang CC, Lurmann F, Berhane K | A longitudinal cohort study of body mass index and childhood exposure to secondhand tobacco smoke and air pollution: The Southern California Children’s Health Study | 2015 | 123 | 117 | 160 | **35.77** |
| Tielsch JM, Katz J, Thulasiraj RD, Coles CL, Sheeladevi S, Yanik EL, Rahmathullah L | Exposure to indoor biomass fuel and tobacco smoke and risk of adverse reproductive outcomes, mortality, respiratory morbidity and growth among newborn infants in south India | 2009 | 120 | 113 | 183 | **22.71** |
| Lam TH, Leung GM, Ho LM | The effects of environmental tobacco smoke on health services utilization in the first eighteen months of life | 2001 | 112 | - | 148 | **11.3** |
| Tarvis MT, Russell MAH, Feyerabend C, Eiser T, Morgan M, Gammage P, Gray EM | Passive exposure to tobacco smoke: Saliva cotinine concentrations in a representative population sample of non-smoking schoolchildren | 1985 | 111 | - | 179 | **7.43** |
| Haustein KO | Cigarette smoking, nicotine and pregnancy | 1999 | 110 | - | 162 | **10.88** |
| Wakefield M, Banham D, Martin J, Ruffin R, McCaul K, Badcock N | Restrictions on smoking at home and urinary cotinine levels among children with asthma | 2000 | 108 | - | 145 | **10.54** |
| Kishi R, Kobayashi S, Ikeno T, Araki A, Miyashita C, Itoh S, Sasaki S, Okada E, Kobayashi S, Kashino I, Itoh K, Nakajima S | Ten years of progress in the Hokkaido birth cohort study on environment and children's health: Cohort profile - Updated 2013 | 2013 | 104 | 103 | 128 | **24.21** |
| Vrijheid M, Casas M, Bergström A, Carmichael A, Cordier S, Eggesbø M, Eller E, Fantini MP, Fernández MF, FernándezSomoano A, Gehring U, Grazuleviciene R, Hohmann C, Karvonen AM, Keil T, Kogevinas M, Koppen G, Krämer U, Kuehni CE, Magnus P, Majewska R, Andersen AMN, Patelarou E, Petersen MS, Pierik FH, Polanska K, Porta D, Richiardi L, Santos AC, Slama R, Sram RJ, Thijs C, Tischer C, Toft G, Trnovec T, Vandentorren S, Vrijkotte TGM, Wilhelm M, Wright J, Nieuwenhuijsen M | European birth cohorts for environmental health research | 2012 | 104 | 91 | 146 | **23.36** |
| Irvine L, Crombie IK, Clark RA, Slane PW, Feyerabend C, Goodman KE, Cater I J | Advising parents of asthmatic children on passive smoking: Randomised controlled trial | 1999 | 101 | - | 168 | **10.76** |
| Blackburn C, Spencer N, Bonas S, Coe C, Dolan A, Moy R | Effect of strategies to reduce exposure of infants to environmental tobacco smoke in the home: Cross sectional survey | 2003 | 100 | 85 | 149 | **13.2** |
| Koch A, Mølbak K, Homøe P, Sørensen P, Hjuler T, Olesen ME, Pejl J, Pedersen FK, Olsen OR, Melbye M | Risk factors for acute respiratory tract infections in young Greenlandic children | 2003 | 99 | 92 | 213 | **16.31** |
| Kirkwood BR, Gove S, Rogers S, LobLevyt J, Arthur P, Campbell H | Potential interventions for the prevention of childhood pneumonia in developing countries: A systematic review | 1995 | 99 | - | 181 | **9.65** |
| Wu F, Takaro TK | Childhood asthma and environmental interventions | 2007 | 97 | - | 172 | **15.82** |
| Eskenazi B, Trupin LS | Passive and active maternal smoking during pregnancy, as measured by serum cotinine, and postnatal smoke exposure II Effects on neurodevelopment at age 5 years | 1995 | 96 | - | 141 | **8.17** |
| Jaakkola JJK, Nafstad P, Magnus P | Environmental tobacco smoke, parental atopy, and childhood asthma | 2001 | 94 | - | 143 | **10.3** |
| Neas LM, Dockery DW, Ware JH, Spengler JD, Ferris BG, Speizer FE | Concentration of indoor particulate matter as a determinant of respiratory health in children | 1994 | 93 | - | 155 | **8.26** |
| Vardavas CI, Hohmann C, Patelarou E, Martinez D, Henderson AJ, Granell R, Sunyer J, Torrent M, Fantini MP, Gori D, AnnesiMaesano I, Slama R, Duijts L, De Jongste JC, Aurrekoetxea JJ, Basterrechea M, Morales E, Ballester F, Murcia M, Thijs C, Mommers M, Kuehni CE, Gaillard EA, Tischer C, Heinrich J, Pizzi C, Zugna D, Gehring U, Wijga A, Chatzi L, Vassilaki M, Bergström A, Eller E, Lau S, Keil T, Nieuwenhuijsen M, Kogevinas M | The independent role of prenatal and postnatal exposure to active and passive smoking on the development of early wheeze in children | 2016 | 92 | 83 | 132 | **31.45** |
| Sun H, Chen W, Wang D, Jin Y, Chen X, Xu Y | The effects of prenatal exposure to low-level cadmium, lead and selenium on birth outcomes | 2014 | 90 | 87 | 119 | **23.8** |
| Jaakkola JJK, Jaakola MS | Effects of environmental tobacco smoke on the respiratory health of children | 2002 | 87 | 76 | 153 | **12.05** |
| Dinas PC, Koutedakis Y, Flouris AD | Effects of active and passive tobacco cigarette smoking on heart rate variability | 2013 | 86 | 77 | 159 | **24.60** |
| Kim S, Arora M, Fernandez C, Landero J, Caruso J, Chen A | Lead, mercury, and cadmium exposure and attention deficit hyperactivity disorder in children | 2013 | 85 | 73 | 135 | **22.21** |
| Norbäck D, Lu C, Zhang Y, Li B, Zhao Z, Huang C, Zhang X, Qian H, Sun Y, Wang J, Liu W, Sundell J, Deng Q | Sources of indoor particulate matter (PM) and outdoor air pollution in China in relation to asthma, wheeze, rhinitis and eczema among pre-school children: Synergistic effects between antibiotics use and PM10 and second hand smoke | 2019 | 83 | 81 | 98 | **41.6** |
| Baxi R, Sharma M, Roseby R, Polnay A, Priest N, Waters E, Spencer N, Webster P | Family and carer smoking control programmes for reducing children's exposure to environmental tobacco smoke | 2014 | 82 | 110 | 452 | **57.06** |
| Norbäck D, Lu C, Wang J, Zhang Y, Li B, Zhao Z, Huang C, Zhang X, Qian H, Sun Y, Sundell J, Deng Q | Asthma and rhinitis among Chinese children — Indoor and outdoor air pollution and indicators of socioeconomic status (SES) | 2018 | 80 | 75 | 92 | **32.83** |
| Farber HJ, Groner J, Walley S, Nelson K | Protecting children from tobacco, nicotine, and tobacco smoke | 2015 | 79 | 61 | 109 | **23.12** |
| Kabir Z, Connolly GN, Alpert HR | Secondhand smoke exposure and neurobehavioral disorders among children in the United States | 2011 | 78 | 76 | 118 | **17.02** |
| Spengler JD, Jaakkola JJK, Parise H, Katsnelson BA, Privalova LI, Kosheleva AA | Housing Characteristics and Children's Respiratory Health in the Russian Federation | 2004 | 78 | 69 | 131 | **11.6** |
| Duramad P, Tager IB, Holland NT | Cytokines and other immunological biomarkers in children's environmental health studies | 2007 | 76 | 70 | 112 | **12.42** |
| Eskenazi B, Bergmann JJ | Passive and active maternal smoking during pregnancy, as measured by serum cotinine, and postnatal smoke exposure I Effects on physical growth at age 5 years | 1995 | 76 | - | 121 | **6.79** |
| Prokhorov AV, Winickoff JP, Ahluwalia JS, OssipKlein D, Tanski S, Lando HA, Moolchan ET, Muramoto M, Klein JD, Weitzman M, Ford KH | Youth tobacco use: A global perspective for child health care clinicians | 2006 | 75 | 60 | 161 | **14.22** |
| Zhou S, Rosenthal DG, Sherman S, Zelikoff J, Gordon T, Weitzman M | Physical, behavioral, and cognitive effects of prenatal tobacco and postnatal secondhand smoke exposure | 2014 | 74 | 69 | 125 | **22.2** |
| Irvine L, Crombie IK, Clark RA, Slane PW, Goodman KE, Feyerabend C, Cater JI | What determines levels of passive smoking in children with asthma? | 1997 | 74 | - | 115 | **7** |
| Ferrante G, Simoni M, Cibella F, Ferrara F, Liotta G, Malizia V, Corsello G, Viegi G, La Grutta S | Third-hand smoke exposure and health hazards in children | 2013 | 73 | - | 138 | **19.18** |
| Stoddard JJ, Gray B | Maternal smoking and medical expenditures for childhood respiratory illness | 1997 | 72 | 62 | 118 | **7.8** |
| Qiu J, He X, Cui H, Zhang C, Zhang H, Dang Y, Han X, Chen Y, Tang Z, Zhang H, Bai H, Xu R, Zhu D, Lin X, Lv L, Xu X, Lin R, Yao T, Su J, Liu X, Wang W, Wang Y, Ma B, Liu S, Huang H, Lerro C, Zhao N, Liang J, Ma S, Ehrenkranz RA, Liu Q, Zhang Y | Passive smoking and preterm birth in Urban China | 2014 | 71 | 61 | 98 | **18.93** |
| Williams SA, Kwan SYL, Parsons S | Parental Smoking Practices and Caries Experience in Pre-School Children | 2000 | 71 | - | 130 | **8.37** |
| Tang D, Warburton D, Tannenbaum SR, Skipper P, Santella RM, Cereijido GS, Crawford FG, Perera FP | Molecular and genetic damage from environmental tobacco smoke in young children | 1999 | 71 | - | 109 | **7.2** |
| Shenassa ED, Brown MJ | Maternal smoking and infantile gastrointestinal dysregulation: The case of colic | 2004 | 69 | 56 | 137 | **11.23** |
| Gilliland FD, Berhane K, Islam T, Wenten M, Rappaport E, Avol E, Gauderman WJ, McConnell R, Peters JM | Environmental tobacco smoke and absenteeism related to respiratory illness in schoolchildren | 2003 | 66 | 56 | 127 | **10.07** |
| Chen Y, Li W, Yu S, Qian W | Chang-ning epidemiological study of children's health: I: Passive smoking and children's respiratory diseases | 1988 | 66 | - | 101 | **4.63** |
| Nafstad P, Kongerud J, Botten G, Hagen JA, Jaakkola JJK | The role of passive smoking in the development of bronchial obstruction during the first 2 years of life | 1997 | 63 | - | 118 | **6.7** |
| Sun Y, Sundell J | Life style and home environment are associated with racial disparities of asthma and allergy in Northeast Texas children | 2011 | 62 | 58 | 86 | **12.87** |
| AlSaleh I, Alsabbahen A, Shinwari N, Billedo G, Mashhour A, AlSarraj Y, Mohamed GED, Rabbah A | Polycyclic aromatic hydrocarbons (PAHs) as determinants of various anthropometric measures of birth outcome | 2013 | 61 | 57 | 80 | **14.54** |
| Gray DM, Turkovic L, Willemse L, Visagie A, Vanker A, Stein DJ, Sly PD, Hall GL, Zar HJ | Lung function in African infants in the Drakenstein child health study impact of lower respiratory tract illness | 2017 | 60 | - | 76 | **19.42** |
| Doherty SP, Grabowski J, Hoffman C, Ng SP, Zelikoff JT | Early life insult from cigarette smoke may be predictive of chronic diseases later in life | 2009 | 60 | 47 | 78 | **10.24** |
| Somerville SM, Rona RJ, Chinn S | Passive smoking and respiratory conditions in primary school children | 1988 | 60 | - | 97 | **4.36** |
| Yi O, Kwon HJ, Kim H, Ha M, Hong SJ, Hong YC, Leem JH, Sakong J, Lee CG, Kim SY, Kang D | Effect of environmental tobacco smoke on atopic dermatitis among children in Korea | 2012 | 59 | 53 | 84 | **13.38** |
| Blount BC, Rich DQ, ValentinBlasini L, Lashley S, Ananth CV, Murphy E, Smulian JC, Spain BJ, Barr D, Ledoux T, Hore P, Robson M | Perinatal exposure to perchlorate, thiocyanate, and nitrate in New Jersey mothers and newborns | 2009 | 58 | 51 | 76 | **10.06** |
| Peters J, Hedley AJ, Wong CM, Lam TH, Ong SG, Liu J, Spiegelhalter DJ | Effects of an ambient air pollution intervention and environmental tobacco smoke on children's respiratory health in Hong Kong | 1996 | 57 | - | 88 | **5.17** |
| Singh GK, Yu SM, Kogan MD | Health, chronic conditions, and behavioral risk disparities among US Immigrant children and adolescents | 2013 | 56 | - | 89 | **13.18** |
| Mishra V, Smith KR, Retherford RD | Effects of cooking smoke and environmental tobacco smoke on acute respiratory infections in young Indian children | 2005 | 56 | 43 | 94 | **8.64** |
| Wang MP, Ho SY, Lam TH | Parental smoking, exposure to secondhand smoke at home, and smoking initiation among young children | 2011 | 55 | 49 | 71 | **10.94** |
| Forsberg B, Pekkanen J, ClenchAas J, Mårtensson MB, St Jernberg N, Bartonova A, Timonen KL, Skerfving S | Childhood asthma in four regions in Scandinavia: Risk factors and avoidance effects | 1997 | 55 | - | 83 | **5.11** |
| Kyu HH, Georgiades K, Boyle M | Maternal smoking, biofuel smoke exposure and child height-for-age in seven developing countries | 2009 | 54 | 57 | 98 | **11.4** |
| Rushton L | Health impact of environmental tobacco smoke in the home | 2004 | 54 | - | 109 | **8.15** |
| Patel DR | Smoking and children | 1999 | 54 | 43 | 118 | **7.45** |
| Svecova V, Rossner Jr P, Dostal M, Topinka J, Solansky I, Sram RJ | Urinary 8-oxodeoxyguanosine levels in children exposed to air pollutants | 2009 | 53 | 50 | 69 | **9.24** |
| Treyster Z, Gitterman B | Second hand smoke exposure in children: Environmental factors, physiological effects, and interventions within pediatrics | 2011 | 52 | - | 89 | **10.84** |
| Polańska K, Hanke W, Gromadzińska J, Ligocka D, Gulczyńska E, Sobala W, Wa̧sowicz W | Polish mother and child cohort study - Defining the problem, the aim of the study and methodological assumptions | 2009 | 52 | 51 | 38 | **7.13** |
| Joad JP | Smoking and pediatric respiratory health | 2000 | 52 | - | 111 | **6.79** |
| Klein J, Koren G | Hair analysis - A biological marker for passive smoking in pregnancy and childhood | 1999 | 51 | 44 | 68 | **5.34** |
| Jung KH, Perzanowski M, Rundle A, Moors K, Yan B, Chillrud SN, Whyatt R, Camann D, Perera FP, Miller RL | Polycyclic aromatic hydrocarbon exposure, obesity and childhood asthma in an urban cohort | 2014 | 49 | 46 | 66 | **13.03** |
| Finkelstein JN, Johnston CJ | Enhanced Sensitivity of the Postnatal Lung to Environmental Insults and Oxidant Stress | 2004 | 49 | 45 | 70 | **6.7** |
| Wang C, Salam MT, Islam T, Wenten M, Gauderman J, Gilliland FD | Effects of in utero and childhood tobacco smoke exposure and β2-adrenergic receptor genotype on childhood asthma and wheezing | 2008 | 48 | - | 85 | **8.31** |
| Schäfer T, Heinrich J, Wjst M, Krause C, Adam H, Ring J, Wichmann HE | Indoor risk factors for atopic eczema in school children from East Germany | 1999 | 48 | - | 71 | **4.76** |
| Lam TH, Chung SF, Betson CL, Wong CM, Hedley AJ | Respiratory symptoms due to active and passive smoking in junior secondary school students in Hong Kong | 1998 | 48 | - | 97 | **5.57** |
| Henderson AJ | The effects of tobacco smoke exposure on respiratory health in school-aged children | 2008 | 47 | 47 | 86 | **9.28** |
| Crombie IK, Wright A, Irvine L, Clark RA, Slane PW | Does passive smoking increase the frequency of health service contacts in children with asthma? | 2001 | 47 | - | 70 | **5.08** |
| Vieira SE | The health burden of pollution: The impact of prenatal exposure to air pollutants | 2015 | 45 | 39 | 73 | **14.55** |
| Miyake Y, Tanaka K, Arakawa M | Active and passive maternal smoking during pregnancy and birth outcomes: The Kyushu Okinawa Maternal and Child Health Study | 2013 | 44 | 44 | 89 | **13.42** |
| Johansson A, Ludvigsson J, Hermansson G | Adverse health effects related to tobacco smoke exposure in a cohort of three-year olds | 2008 | 44 | 40 | 77 | **8.39** |
| Lee BE, Hong YC, Park H, Ha M, Hyeong Kim J, Chang N, Roh YM, Kim BN, Kim Y, Oh SY, Ju Kim Y, Ha EH | Secondhand smoke exposure during pregnancy and infantile neurodevelopment | 2011 | 43 | 40 | 72 | **9.86** |
| Johansson A, Halling A, Hermansson G | Indoor and outdoor smoking: Impact on children's health | 2003 | 43 | 39 | 77 | **6.33** |
| Emmons KM, Wong M, Hammond SK, Velicer WF, Fava JL, Monroe AD, Evans JL | Intervention and policy issues related to children's exposure to environmental tobacco smoke | 2001 | 43 | - | 67 | **4.78** |
| Hwang SH, Hwang JH, Moon JS, Lee DH | Environmental tobacco smoke and children's health | 2012 | 42 | - | 88 | **10.83** |
| Tong EK, England L, Glantz SA | Changing conclusions on secondhand smoke in a sudden infant death syndrome review funded by the tobacco industry | 2005 | 42 | 38 | 74 | **6.77** |
| Perera F, Li TY, Lin C, Tang D | Effects of prenatal polycyclic aromatic hydrocarbon exposure and environmental tobacco smoke on child IQ in a Chinese cohort | 2012 | 41 | 35 | 59 | **9.3** |
