# Supplementary material for: Environmental tobacco smoke and children’s health: a bibliometric and altmetric analysis of 100 most cited articles
Source: BMC Public Health. 2023 Nov 9;23:2208. doi: 10.1186/s12889-023-16242-1 (PMC10634132; doi:10.1186/s12889-023-16242-1)
Supplement: Supplementary file 2 — Supplementary Material 2 [file 12889_2023_16242_MOESM2_ESM.docx]

S2 Table - Journals with individual contribution to the 100 most-cited articles on ETS and CH

| **Journal Name** | **Impact Factor** | **Quartile** | **Subject Area and Category** | **Number of articles** | **Country** |
| --- | --- | --- | --- | --- | --- |
| Pediatrics | 9.703 | 1 | Medicine | 12 | United States |
| American Journal of Epidemiology | 5.363 | 1 | Medicine | 7 | United Kingdom |
| International Journal of Epidemiology | 9.685 | 1 | Medicine | 7 | United Kingdom |
| Environmental Research | 8.431 | 1 | Biochemistry, Genetics and Molecular Biology; Environmental Science | 6 | United States |
| Environmental Health Perspectives | 11.035 | 1 | Environmental Science; Medicine | 5 | United States |
| American Journal of Respiratory and Critical Care Medicine | 30.528 | 1 | Medicine | 4 | United States |
| British Medical Journal | 93.333 | 1 | Medicine | 4 | United Kingdom |
| Acta Paediatrica | 4.056 | 1 | Medicine | 2 | United States |
| American Journal of Public Health | 11.561 | 1 | Medicine | 2 | United States |
| Environment International | 13.352 | 1 | Environmental Science | 2 | United Kingdom |
| Journal of Toxicology and Environmental Health-Part B-Critical Reviews | 8.071 | 1 | Environmental Science; Pharmacology, Toxicology and Pharmaceutics | 2 | United Kingdom |
| Paediatric Respiratory Reviews | 5.526 | 1 | Medicine | 2 | United Kingdom |
| Reviews on Environmental Health | 4.022 | 2 | Environmental Science; Medicine; Social Science | 2 | Germany |
| Science of the Total Environment | 10.753 | 1 | Environmental Science | 2 | Netherlands |
| Thorax | 9.102 | 1 | Medicine | 2 | United Kingdom |
| American Journal of Preventive Medicine | 6.604 | 1 | Medicine | 1 | United States |
| Archives of Disease in Childhood | 4.920 | 2 | Medicine | 1 | United Kingdom |
| BMC Pregnancy and Childbirth | 3.105 | 1 | Medicine | 1 | United Kingdom |
| Biomarkers | 2.633 | 2 | Biochemistry, Genetics and Molecular Biology; Environmental Science; Medicine | 1 | United Kingdom |
| Bulletin of the World Health Organization | 13.831 | 1 | Medicine | 1 | Switzerland |
| Cancer Epidemiology Biomarkers and Prevention | 4.090 | 1 | Medicine | 1 | United States |
| Caries Research | 3.918 | 1 | Dentistry | 1 | Switzerland |
| Chemosphere | 8.943 | 1 | Chemistry; Environmental Science; Medicine | 1 | United Kingdom |
| Clinics in Chest Medicine | 4.967 | 2 | Medicine | 1 | United Kingdom |
| Cochrane Database of Systematic Reviews | 12.008 | 1 | Medicine | 1 | United Kingdom |
| Current Problems in Pediatric and Adolescent Health Care | 2.557 | 2 | Medicine | 1 | United States |
| Environmental Health and Preventive Medicine | 4.395 | 2 | Medicine | 1 | Japan |
| Environmental Science and Technology | 11.357 | 1 | Environmental Science | 1 | United States |
| Epidemiology | 4.860 | 2 | Medicine | 1 | United States |
| European Journal of Pediatrics | 3.860 | 1 | Medicine | 1 | Germany |
| European Journal of Public Health | 4.424 | 1 | Medicine | 1 | United Kingdom |
| European Respiratory Journal | 33.795 | 1 | Medicine | 1 | Switzerland |
| Human and Experimental Toxicology | 3.247 | 2 | Environmental Science; Medicine; Pharmacology, Toxicology and Pharmaceutics | 1 | United States |
| Indian Journal of Pediatrics | 5.319 | 2 | Medicine | 1 | India |
| International Journal of Cardiology | 4.039 | 1 | Medicine | 1 | Ireland |
| International Journal of Clinical Pharmacology and Therapeutics | 0.976 | 3 | Medicine; Pharmacology, Toxicology and Pharmaceutics | 1 | Germany |
| International Journal of Chronic Obstructive Pulmonary Disease | 2.893 | 1 | Medicine; Pharmacology, Toxicology and Pharmaceutics | 1 | United Kingdom |
| International Journal of Environmental Research and Public Health | 4.614 | 1 | Environmental Science; Medicine | 1 | Switzerland |
| International Journal of Occupational Medicine and Environmental Health | 1.828 | 3 | Medicine | 1 | Poland |
| Journal of Epidemiology and Community Health | 6.286 | 1 | Medicine | 1 | United Kingdom |
| Journal of Translational Medicine | 8.440 | 1 | Biochemistry, Genetics and Molecular Biology; Medicine | 1 | United Kingdom |
| Korean Journal of Pediatrics | NA | 1 | Medicine; Nursing | 1 | South Korea |
| Monaldi Archives for Chest Disease | NA | 3 | Medicine | 1 | Italy |
| Mutation Research Fundamental and Molecular Mechanisms of Mutagenesis | 3.151 | 1 | Biochemistry, Genetics and Molecular Biology; Environmental Science | 1 | Netherlands |
| Mutation Research Reviews in Mutation Research | 7.015 | 1 | Biochemistry, Genetics and Molecular Biology; Environmental Science | 1 | Netherlands |
| Nicotine and Tobacco Research | 5.825 | 1 | Medicine | 1 | United Kingdom |
| Pediatric Pulmonology | 4.090 | 1 | Medicine | 1 | United States |
| Population and Environment | 4.283 | 1 | Environmental Science; Social Science | 1 | Netherlands |
| Preventive Medicine | 4.637 | 1 | Medicine | 1 | United States |
| Public Health Reports | 3.117 | 1 | Medicine | 1 | United States |
| Respiratory Research | 7.162 | 1 | Medicine | 1 | United Kingdom |
| Scandinavian Journal of Work Environment and Health | 5.492 | 1 | Medicine | 1 | Finland |
| Tobacco Control | 6.953 | 1 | Medicine; Social Science | 1 | United Kingdom |
| Toxicology Letters | 4.271 | 1 | Medicine; Pharmacology, Toxicology and Pharmaceutics | 1 | Netherlands |

NA - Not available in the 2022 Journal Citation Reports
