# Supplementary material for: Environmental tobacco smoke and children’s health: a bibliometric and altmetric analysis of 100 most cited articles
Source: BMC Public Health. 2023 Nov 9;23:2208. doi: 10.1186/s12889-023-16242-1 (PMC10634132; doi:10.1186/s12889-023-16242-1)
Supplement: Supplementary file 4 — Supplementary Material 4 [file 12889_2023_16242_MOESM4_ESM.docx]

S4 Table - Correlations among the Citations count, AAS and Dimensions count

| Citation count  (Scopus) | 1 |  |  |  |  |  |
| --- | --- | --- | --- | --- | --- | --- |
| Citation count  (Web of Science) | 0.82 | 1 |  |  |  |  |
| Citation count  (Google Scholar) | 0.95 | 0.78 | 1 |  |  |  |
| Mean citation  density | 0.74 | 0.82 | 0.75 | 1 |  |  |
| AAS | 0.17 | 0.27 | 0.17 | 0.52 | 1 |  |
| Dimensions count | 0.98 | 0.84 | 0.94 | 0.79 | 0.24 | 1 |
|  | Citation count  (Scopus) | Citation count  (Web of Science) | Citation count  (Google Scholar) | Mean citation  density | AAS | Dimensions count |
